# Study of plasmid mediated quinolone resistance genes among *Escherichia coli* and *Klebsiella pneumoniae* isolated from pediatric patients with sepsis

**DOI:** 10.1038/s41598-024-61357-z

**Published:** 2024-05-24

**Authors:** Ahmed Gomaa Elsayed, Ehab M Fahmy, Mona Abdellatif Alsayed, Mai Essam Ahmed, Maysaa El Sayed Zaki, Mohamed Mofreh Mohamed

**Affiliations:** 1 Medical Microbiology and Immunology, Mansoura Faculty of Medicine, Mansoura, Egypt; 2 Medical Microbiology and Immunology, Helwan Faculty of Medicine, Helwan, Egypt; 3 Mansoura Faculty of Medicine, Mansoura, Egypt; 4 Clinical Pathology, Beni suef Faculty of Medicine, Beni Suef, Egypt; 5 Clinical Pathology, Mansoura Faculty of Medicine, Mansoura, Egypt

**Keywords:** Sepsis. Pediatrics, *PMQ,*, *qnrA*, *qnrB*, *qnrS*, PCR, Genetics, Microbiology

## Abstract

The resistance to antibiotics in Gram-negative bacilli causing sepsis is a warning sign of failure of therapy. *Klebsiella pneumoniae* (*K*. *pneumoniae*) and *Escherichia coli* (*E. coli*) represent major Gram-negative bacilli associated with sepsis. Quinolone resistance is an emerging resistance among *E. coli* and *K. pneumoniae*. Therefore, the present study aimed to study the presence of plasmid-mediated quinolone resistance (*PMQR*) genes *qnrA, qnrB,* and *qnrS* by polymerase chain reaction (PCR) in *E. coli* and *K. pneumoniae* isolated from pediatric patients with sepsis. This was a retrospective cross-sectional study that included pediatric patients with healthcare-associated sepsis. The *E. coli* and *K. pneumoniae* isolates were identified by microbiological methods. *PMQR* genes namely *qnrA, qnrB,* and *qnrS* were detected in *E*. *coli* and *K. pneumoniae* isolates by PCR. The results were analyzed by SPPS24, and the qualitative data was analyzed as numbers and percentages and comparison was performed by Chi-square test, P was significant if < 0.05. The most prevalent gene detected by PCR was *qnrA* (75%), followed by *qnrB* (28.1%), and *qnrS* (25%). The most frequently detected *qnr* gene in *E coli* and *K. pneumoniae* was *qnrA* (28.8%, and 16.3% respectively). The present study highlights the high prevalence of ciprofloxacin resistance among *E. coli* and *K. pneumoniae* isolated from pediatric patients with healthcare-associated sepsis. There was a high frequency of *PMQR* genes in *E. coli* and *K. pneumoniae* isolated from pediatric patients. Therefore, it is important to monitor the spread of *PMQR* genes in clinical isolates to ensure efficient antibiotic use in those children. The finding denotes the importance of an antibiotics surveillance program.

## Introduction

Sepsis in pediatric patients is associated with marked morbidity and mortality in high- and low-income countries. Children are susceptible to this infection due to the immaturity of the immune system, the presence of comorbidities, and frequent contact with healthcare teams^1^. Severe sepsis and septic shock are responsible for 10–25% of Pediatric Intensive Care Unit (PICU) admissions worldwide^2,3^. Sepsis morbidity and mortality affect children differently around the world^2–4^. Although international data shows a mortality of 25% and the presence of long-term sequelae in 20% of survivors, it is known that children living in low- and middle-income countries face the highest burden^2^ The antibiotic therapy for this condition depends upon the primary site of the infection, the presence of comorbidities, and the history of antibiotic exposure. The preferred method of antibiotic administration is intravenously or intramuscularly^5^.

The common pathogen associated with healthcare sepsis is Gram-negative bacilli^6–8^. These pathogens develop antimicrobial resistance rapidly after misuse of antibiotics, lack of adherence to infection control measures, and the capacity of the organisms to develop antibiotic resistance through a wide range of mechanisms^9–11^. Antibiotic resistance leads to increased hospital costs with poor clinical outcomes^6^.

*Escherichia coli* (*E.coli*) and *Klebsiella pneumoniae* (*K. pneumoniae*) had an important role in antibiotic resistance due to the carriage of antibiotics resistance genetic determinant regions and their transport to other *Enterobacteriaceae* species^12,13^. The transfer of antibiotics resistance genetic determinant regions is mediated via plasmids and horizontal gene transfer^14^. In different countries, the resistance to antibiotics of *E. coli* and *K. pneumoniae* has been reported in the range of 85%-90% by the World Health Organization^15^.

Fluoroquinolone antibiotics like ciprofloxacin are broad antibiotics used effectively in the eradication of different bacterial pathogens^16^. However, with the emergence of resistance to this antibiotic, there is a reduction in therapeutic options for *E. coli Klebsiella pneumoniae* and other *Enterobacteriaceae* species^17^. Some guidelines approve the use of quinolones in pediatrics when the first line of treatment is not effective and no alternative is available^18,19^. Normally, the quinolone of choice is ciprofloxacin, due to its higher safety profile in comparison with other quinolones^20^. There are two mechanisms for the development of quinolone resistance, either through chromosomal mutation or through plasmid-mediated quinolone resistance (*PMQR*). Chromosomal mutations lead to the alteration of target enzymes and the affinity of drugs binding to them. The chromosomal mutations occur in the genes in the quinolone resistance determining regions **(QRDRs).** Plasmid-mediated quinolone resistance is mediated through the DNA gyrase protection and topoisomerase IV from the activity of quinolone antibiotics through the qnr genes namely*,* qnrA*,* qnrB*,* qnrC*,* qnrD*,* and *qnrVC*^21^*.* There is also involvement of the aminoglycoside-modifying enzyme through the gene aac*(6’)-Ib-cr* by the acetylation of fluoroquinolones which leads to reduced susceptibility to quinolone antibiotics^21^. The other mechanism of quinolone resistance is the enhancement of the efflux pump activity facilitated by quinolone efflux pump *(qepA)* and *oqxAB* leading to the reduction in the susceptibility to quinolones and increased extended-spectrum beta-lactamase activity^22^.

The *qnr* genes lead to ciprofloxacin resistance by encoding proteins protecting DNA gyrase and topoisomerase IV from inhibition by quinolones^23^. The action of *PMQR* mutations is through the involvement of a modified enzyme that adds acetyl group C7 piperazine ring found in quinolones such as ciprofloxacin. This modification leads to the reduction of the drug's activity and confers resistance to quinolones^23^.

There are few reports about the emergence of quinolone resistance in *E. coli* and *K. pneumoniae* from pediatric patients to quinolone^24,25^. However, there are limited data about the resistance of *Klebsiella pneumoniae* and *E. coli* to ciprofloxacin from children with sepsis in Egypt.

Therefore, the present study aimed to study the presence of *PMQR* genes *qnrA, qnrB*, and *qnrS* by polymerase chain reaction (PCR) in *E. coli* and *K. pneumoniae* isolated from children with sepsis.

## Method

This was a retrospective cross-sectional study including pediatric patients with healthcare-associated sepsis recruited from Mansoura University Children's Hospital, Egypt from January 2020 to January 2023. The included children were complaining of sepsis according to the criteria of the Center for Disease Control^7^ with isolated bacterial pathogens *Escherichia coli* and *Klebsiella pneumoniae* by microbiological laboratory culture. Children with community-acquired sepsis or children with other pathogens associated with sepsis by microbiological culture were excluded from the study. The study was approved by the Mansoura Faculty of Medicine ethical committee (R.22.10.2354) and written approval was obtained from the parents of each child.

### Microbiological culture

Five -milliliter blood samples were taken from each child and inoculated to the bottles of the blood culture system Bact/alert^®^ 3D (bioMérieux– France). Blood culture bottles found positive were subculture on MacConkey agar plates and blood agar plates at 37 °C for 24 h. Colonies were subjected to identification by Gram stain and further identification by Vitek 2 system (bioMérieux–France). Identified isolates K. pneumoniae and Escherichia coli were subjected to further studies for antibiotics sensitivity by disc diffusion method and molecular study by PCR for detection of *PMQR* genes.

### Antibiotics sensitivity

Isolated *Escherichia coli* and *K pneumoniae* were tested for antibiotic sensitivity by the antibiotics disc diffusion method. The used discs were Levofloxacin (30 µg), Amoxicillin (30 µg), Cefotaxime (30 µg), Gentamicin (10 µg), Ciprofloxacin (5 µg), Tetracycline (10 µg), Gatifloxacin(10 µg), Norfloxacin (30 µg), Ceftriaxone (10 µg). The method used and the interpretation of the results were according to the Clinical Laboratory Standards Institute, 2018 (CLSI)^26^ Multi-drug resistance (MDR) was identified as resistance to three or more antibiotics classes.

### Detection of resistance for ciprofloxacin by Broth Microdilution method (BMD)

*E. coli* and *K. pneumoniae* resistant to ciprofloxacin antibiotic disc were then tested for ciprofloxacin resistance by BMD method following the instruction of CLSI,2018^26^ to identify the minimal inhibitory concentration (MIC). The isolates were suspended in 0.1 ml of Muller-Hinton broth and serial preparations of ciprofloxacin from 0.25 µg/ml to 128 µg/ml were added and incubation was performed at 37 °C for 20 h. Pure bacterial suspension in broth was used as positive control without adding ciprofloxacin. The well with the highest dilution with no turbidity was determined as MIC and the wells were sub-cultured to determine the minimal bactericidal concentration^17^. The interpretation was according to CLSI guidelines^17^ at the following concentrations, ciprofloxacin susceptible with MIC ≤ 1 μg/mL, intermediate with MIC 2 μg/mL. Resistant with MIC ≥ 4 μg/mL E. coli ATCC 25922 was used as positive control.

### PCR for detection of PMQR genes qnrA, qnrB, and plasmid DNA extraction

Plasmid DNA purification was done using a commercial kit for plasmid DNA purification NucleoSpin^®^ Plasmid EasyPure (item 740727.50- Macherey–Nagel-Germany). The method was used according to the manufacturer's instructions. The extracted plasmid DNA was kept frozen at – 20 °C till further amplification by PCR.

#### PCR

The used primers for the amplification of *qnrA, qnrB,* and *qnrS* genes were summarized in Table 1. The amplification mixture was a ready-to-use reagent mixture DreamTaq Green PCR Master Mix (Thermofisher Scientific, USA).

**Table 1:** The primers used with their sequences, the size of the amplified product base pair (bp), and the annealing temperature.

Gene	Primer	Product size (bp)	Annealing temp. (°C)
*qnr A*	5’-TCAGCAAGAGGATTTCTCA-3 5’-GGCAGCACTATTA CTCCCA-3	516	48°C
*qnr B*	5’-ATGACGCCATTACTGTATAA3′ 5’-GATCGCAATGTGTGAAGT TT-3	562	53°C
*qnr S*	5’-CAATCATACATATCGGCACC-3 5’-TCAGGATAAACAACAATACCC-3	369	60°C

Amplification procedures were performed with the following conditions: initial denaturation at 94 °C for 5 min, followed by 35 cycles of denaturation at 94 °C for 30 s, (different annealing temperatures according to Table 1) for 30 s with an extension at 72 °C for 50 s, and a final extension for one cycle at 72 °C for 5 min in thermal cycler (Gene Amp PCR System 9700/Perkin Elmer Corporation, Norwal CT/USA)^27^.

### Statistical analysis

The results of the study were analyzed by the SPPS22.0. Qualitative data was expressed as numbers and percentages and these data were compared using Chi-square and P was considered significant if < 0.05. The age was expressed as median, minimum, and maximum values as it was nonparametric.

### Ethics approval and consent to participate

All methods were performed by the ethical standards as laid down in the Declaration of Helsinki and its later amendments or comparable ethical standards. The ethical approval of the study was obtained from the ethical committee of Mansoura Faculty of Medicine (R.22.10.2354) and written Informed consent was obtained from the parent of each child.

## Results

The study included 52 (48.6%) males and 57(51.4%) females with ages ranging from two years to 15 years, median 8.00 years. Renal disorders (42. 1%) followed by Diabetes mellitus (32.7%) were associated with sepsis. The associated devices with sepsis were mainly urinary catheters (37.4%) followed by peripheral venous catheter (34.6%) and central venous catheters (28.03%), Mean ± SD days of stay in ICU was 6.34 ± 2.15 days. The outcome of the sepsis was recovery in 97 patients (90.7%) and death in 10 patients (9.3%), Table 2. All children received empirical therapy of aminoglycosides plus beta-lactams antibiotics, data not shown.

**Table 2:** Basic demographic and clinical data of the studied children.

Data	No. (%)
Age (years)	
Minimum	2
Maximum	15
Median	8.00
Sex	
Male (No.-%)	52 (48.6%)
Female(No.-%)	55 (51.4%)
Comorbidities	
Diabetes mellitus (No.-%)	35 (32.7%)
Renal disorders (No.-%)	45 (42.1%)
Malignancy (No.-%)	10 (9.3%)
Pneumonia (No.-%)	7 (6.5%)
Surgery (No.-%)	10 (9.3%)
Associated device	
Urinary catheter (No.-%)	40 (37.4%)
Central venous catheter (No.-%)	30 (28.03%)
Peripheral venous catheter(No.-%)	37 (34.6%)
Outcome	
Recovery	97 (90.7%)
Death	10 (9.3%)
ICU duration of stay (mean ± SD)	6.34 ± 2.15

### The isolated E. coli and K. pneumoniae had high resistance to gentamicin (50.5%), levofloxacin (43.9%), amoxicillin (40.2%), and ciprofloxacin (36.4%)

The MDR was detected in 44.8% of the total isolates, with MDR of *E. coli* 42.3% and MDR of *K. pneumoniae* 47.3%, (Table 3).

**Table 3:** Antibiotic resistance of the isolated *E.coli* versus *K. pneumoniae.*

	Total (n = 107)	*E, coli* (n = 52)	*K. pneumonia* (n = 55)	P
	N. (%)	N. (%)	N. (%)	
Ciprofloxacin	39 (36.4)	15 (28.8)	24 (43.6)	0.16
Amoxicillin	43 (40.2)	20 (38.5)	23 (41.8)	0.35
Gatifloxacin	32 (29.9)	14 (26.9)	18 (32.7)	0.78
Tetracycline	30 (28,03)	11 (21.2)	19 (34.5)	0.3
Ceftriaxone	22 (20.6)	10 (19.2)	12 (21.8)	0.2
Norfloxacin	35 (32.7)	24 (46.2)	31 (56.4)	0.57
Levofloxacin	47 (43.9)	21 40.4	26 (47.3)	0.71
Cefotaxime	28 (26.2)	16 (30.8)	12 (21.8)	0.47
Gentamicin	54 (50.5)	26 (50)	28 (50.9)	0.5
MDR	48 (44.8)	22 (42.3)	26 (47.3)	0.37

Thirty-nine isolates were resistant to ciprofloxacin by disc diffusion method with 32 (82.1%) isolates being resistant to ciprofloxacin by MIC method. All the isolates resistant to ciprofloxacin by MIC were positive for *PMQR* genes by PCR, (Table 4).

**Table 4:** Phenotypic resistant isolates to ciprofloxacin and genotypic resistance by PCR.

	Resistant Isolates by ciprofloxacin antibiotic disc (n = 39)
	N. (%)
Resistant bacteria for ciprofloxacin by MIC	32 (82.1)
Positive PCR for *PQMR* genes	32 (82.1)

The most frequent gene detected by PCR was *qnrA* (75%), followed by *qnrB* (28.1%), and *qnrS* (25%). All genes were detected in two isolates (6.2%), the presence of *qnrA* and *qnrB* genes was detected in three isolates (9.4%), and *qnrA* plus *qnrS* was detected in 2 isolates (6.2%), (Table 5).

**Table 5:** Frequency of *qnrA, qnrB* and *qnrS* genes among isolates.

	Positive isolates by PCR (n = 32)
*qnrA*	24 (75%)
*qnrB*	9 (28.1%)
*qnrS*	8 (25%)
*qnrA* + *qrB*	3 (9.4%)
*qnrA* + *qrB* + *qrS*	2 (6.2%)
*qnrA* + *qrS*	2 (6.2%)

The clinical isolates were *E. coli* from 52 of pediatric patients with sepsis and 55 *K. pneumoniae* from pediatric patients with sepsis. The most frequently detected *qnr* gene in *Escherichia coli* and *Klebsiella pneumoniae* was *qnrA* (28.8%, and 16.3% respectively). There was an insignificant difference between the frequency of positive PCR between *E. coli* and *K. pneumonia* (P = 0.11), and an insignificant difference in the frequency of *qnrA, qnrB*, and *qnrS* genes (P = 0.09, P = 0.54, P = 0.33 respectively), (Table 6).

**Table 6:** Frequency of *PMQR* genes in E.coli versus K. pneumonia.

	E.coli (n = 52)	K.pnumoniae (n = 55)	P
	N. %	N. %	
PCR positive	19 (36.5)	13 23.6)	0.11
*qrA*	*15 *(*28.8*)	*9 *(*16.3*)	*0.09*
*qrB*	*4 *(*7.7*)	*5 *(*9.1*)	*0.54*
*qrS*	*5 *(*9.6*)	*3 *(*5.4*)	*0.33*

Significants values are in italic.

The MDR resistance and the presence of *qnr* genes had insignificant associations (P = 0.53), but data was not shown.

The study of association between demographic data and clinical data with resistant isolates for ciprofloxacin by MIC revealed insignificant association between male and female (P = 0.86), age (P = 0.33), insignificant association between the presence of different comorbidities and resistant isolates (P = 0.98), insignificant association between the presence of urinary catheter, central venous catheter and peripheral venous catheter (P = 0.87) and insignificant association between ICU stay days and the presence of resistant isolates (P = 0.4). There was an insignificant association between the outcome of sepsis and the presence of resistant isolates (P = 0.1970. There was a significant association between PCR and resistance to ciprofloxacin by MIC all isolates positive by PCR were resistant by MIC (P = 0.001), Table 7

**Table 7:** Association between demographic, clinical data and PCR with resistant isolates to ciprofloxacin by MIC.

	Ciprofloxacin resistant by MIC (n = 32)	P
	N. %	
Sex		
Male	15 (46.9)	0.86
Female	17 (53.1)	
Age (mean ± SD)	7.62 ± 3.92	0.33
Comorbidities		
DM	11 (34.4)	0.98
Renal disorders	15 (46.9)	
Malignancy	3 (9.4)	
Surgical	2 (6.3)	
Pneumonia	1 3.1	
Urinary catheter	10 (31.3)	0.87
Central venous catheter	9 (28.1)	
Peripheral venous catheter	13 (40.6)	
Days of ICU (mean ± SD)	6.78 ± 2.55	0.4
Outcome		
Death	2 (6.3)	0.197
Recovery	30 (93.7)	
PCR genes	32 (100)	0.001

## Discussion

Pediatric patients admitted to hospitals are susceptible to healthcare-associated infections due to immature immune systems^28^. Bloodstream infections are among the most common infections^29^.

In the present study, *E*. *coli* and *K*. *peumoniae* were common Gram-Negative bacilli isolated from pediatric patients. This finding was reported previously^28–31^. There were medical devices associated with infections in those children such as peripheral venous catheters, urinary catheters, and central venous catheters. This finding indicates the necessity of proper implementation of infection control guidelines and early removal of medical devices to ensure minimal healthcare-associated infections^30^.

The children in the present study were complaining mainly of Diabetes mellitus and renal disorders as underlying comorbidities. The admission etiologies in developing countries are associated with medical conditions rather than surgical conditions in patients with young age^32^.

The isolated *E. coli* and *K. pneumoniae* had high resistance to gentamicin (50.5%), levofloxacin (43.9%), amoxicillin (40.2%), and ciprofloxacin (36.4%). The results were in line with previous studies with high resistance to amoxicillin and ciprofloxacin^31,33^. The aminoglycosides such as gentamycin are usually used as the second choice combined with penicillin for the treatment of severe hospital-acquired infections as sepsis. Thus, this prescription can be responsible for the high resistance to gentamicin reported in the present study and previously^34^. The MDR was detected in 44.8% of the total isolates. This high percentage of multiple antibiotics resistance is a common finding worldwide among Gram-negative bacilli associated with healthcare-associated infections^31^.

Most resistant isolates to ciprofloxacin by disc diffusion method were resistant to ciprofloxacin by MIC method. The results of the disc diffusion method agree with MIC in the detection of resistant isolates to ciprofloxacin^35^.

Ciprofloxacin-resistant *K. pneumoniae* was (43.6%) and ciprofloxacin-resistant *E. coli* was (28.8%). A higher rate of resistant E. coli (64.4%) to ciprofloxacin was reported in a previous study^36^. The resistance rates differ according to the difference in the geographical regions due to the difference in the prescription of ciprofloxacin and due to the difference in the laboratory methods used in the reporting of the resistance^37^.

In the present study, all the resistant isolates for ciprofloxacin were positive for the *PMQR* genes. In a previous study, 80% of resistant isolates of *E*. *coli* and *Klebsiella pneumoniae* were positive for *PMQR* genes^38^. The high ciprofloxacin resistance among the isolates may be attributed to the selection mediated by other antibiotics such as cephalosporins leading to the selection of isolates carrying *PMQR* genes as previously reported^39^. In the present study, all isolates resistant to ciprofloxacin by MIC method were positive by PCR for *PMQR* genes, this finding supports the usefulness of the phenotypic resistant isolates to ciprofloxacin by MIC as a screening method due to its relatively low cost compared to molecular techniques.

The most frequent gene detected by PCR was *qnrA* (75%), followed by *qnrB* (28.1%), and *qnrS* (25%). On contrary to previous studies reporting that the prevalence of *qnr* genes ranged from 2% up to 40% in previous studies^40,41^^,^^38,42^. In Egypt, the rate of positive *qnr* genes in clinical isolates was 56.8%^43^. However, this difference in the rates of *qnr* gene detection may be attributed to the geographical difference, the difference in the type of infections of the studied population and their ages, and the difference in the prescription of antibiotics system.

The most prevalent gene was *qnrA* in both *E. coli* and *K. pneumoniae*. This finding was like the previous report including samples from blood culture specimens^44^ On the contrary previous study reported that the *qnrB* gene was predominant in *K*. *pneumoniae* isolates^42^.

All *qnr* genes were present in two isolates (6.2%), the presence of *qnrA* and *qnrB* genes was detected in three isolates (9.4%), and *qnrA* plus *qnrS* was detected in two isolates. The compound presence of *PMQR* genes leads to the selection of resistant mutants^45^.

Ciprofloxacin can be used combined with beta-lactams antibiotics as a second line of therapy in sepsis if there was limited therapeutic response to the combined aminoglycosides with beta-lactams^46^.

The limitation of the study includes the need for more investigations including different resistance genes or virulence factor genes by different laboratory methods.

## Conclusion

There was a high frequency of ciprofloxacin resistance among *Escherichia coli* and *Klebsiella pneumoniae* isolated from pediatric patients with healthcare-associated sepsis. There was a high prevalence of *PMQR* genes among *E. coli* and *K*. *pneumoniae* isolated from pediatric patients. Therefore, it is important to monitor the spread of *PMQR* genes in clinical isolates to ensure efficient antibiotic use in those children. The finding denotes the importance of an antibiotics surveillance program.

## Data Availability

The datasets generated and analyzed during the current study are available in the Fighshare repository at. https://doi.org/10.6084/m9.figshare.24615552.v1.

## Abbreviations

*E.coli*: *Escherichia coli*
*K. pneumonia*: *Klebsiella pneumoniae*
PCR: Polymerase chain reaction
